# Bleeding and thrombotic complications and their impact on mortality in patients supported with left ventricular assist device for cardiogenic shock

**DOI:** 10.1177/02676591221127651

**Published:** 2022-09-23

**Authors:** Ingrid Bekono-Nessah, Alex Rosenburg, Christopher T Bowles, Fernando Riesgo-Gil, Ulrich Stock, Richard R Szydlo, Mike Laffan, Deepa J Arachchillage

**Affiliations:** 1 Centre for Haematology, Department of Immunology and Inflammation, 4615 Imperial College London, London, UK; 2 Department of critical care, 4964 Royal Brompton & Harefield Hospitals, Part of Guy’s & St Thomas’ NHS Foundation Trust, London, UK; 3 Department of Cardiothoracic Transplantation and Mechanical Circulatory Support, 4964 Royal Brompton & Harefield Hospitals, Part of Guy’s & St Thomas’ NHS Foundation Trust, London, UK; 4 Department of Haematology, 4964 Royal Brompton & Harefield Hospitals, London, UK

**Keywords:** left ventricular assist device, bleeding, thrombosis, anaemia, mortality

## Abstract

**Introduction:**

Thrombosis and bleeding are major complications in patients supported with left ventricular assist devices (LVADs). We aimed to assess the incidence of bleeding and thrombosis in patients supported with a HeartWare left ventricular assist device (HVAD), their predictive factors and their impact on mortality.

**Methods:**

A single centre retrospective observational study of patients supported with HVAD over 5 years from January 2015 to October 2020.

**Results:**

A total 139 patients (median age 52.5, 72.1% male) were included for analysis. The probability of 1-year survival was 73.1%. Advanced age (>60 years) and EuroSCORE II score (>20%) were independently associated with reduced survival. Major bleeding and thrombosis occurred in 46.8% and 35.3% respectively. Secondary mechanical circulatory support (MCS) increased likelihood of experiencing major bleeding (HR: 2.76, 95%1.65–4.62, *p* < 0.0001) whilst patients receiving aspirin were protected from bleeding and thrombosis (HR: 0.34 95% CI 0.19–0.58, *p* < 0.001). Pre-operative anaemia (HR: 3.02, 95% CI: 1.6–5.7, *p* = 0.014) and use of a secondary MCS device (HR: 2.78, 95% CI: 1.2–6.3, *p* = 0.001) were associated with an increased risk of thrombosis. Patients with any major bleeding (with or without thrombosis) had a 7.68-fold (95% CI 3.5–16.8) increased risk of death compared to those without. In contrast, ‘thrombosis only’ patients had 4.23-fold (95% CI 1.8–10.2) increased risk of death compared to those without thrombosis. The risk of mortality was increased in patients with any thrombosis and the risk of death was highest in patients with major bleeding and thrombosis (HR: 16.49 [95% CI 7.7–35.3]).

**Conclusions:**

Major bleeding and thrombosis significantly increase the 1-year mortality. Optimal perioperative haemostasis and anticoagulation remains crucial in patients supported with HVAD.

## Introduction

Left ventricular assist devices (LVADs) are used temporarily as a bridge-to-recovery to allow improvement in contractility and reversal of remodelling or long term implantable LVADS as a bridge to transplant, candidacy to transplant, or (in a small percentage) to recovery. Some patients who are not candidates for transplant receive LVADs as destination therapy.^1^ Clinical use of mechanical circular support (MCS) devices has significantly increased, despite their association with major complications, mainly thrombosis and bleeding, and a paucity of evidence supporting their use in cardiogenic shock, leading to a delay in the development of guidelines. Device thrombosis is largely caused by plasma protein adsorption to the artificial surfaces resulting in sequential activation of contact-mediated factors XII (FXII) and XI (FXI).^2^ By reducing the surface area exposed to blood components, third-generation continuous-flow (CF-) LVADs, may reduce contact activation^3,4^ but ischaemic stroke continues to be a major complication. To minimise this risk, device surfaces are heparinised, and use of systemic antithrombotic drugs is mandatory.^5^ On the other hand, patients are also at risk of bleeding which may be aggravated by use of anticoagulant and antiplatelet treatment.^6^ The shear force of the rotary pump facilitates cleavage of von Willebrand factor (VWF) multimers by ADAMTS-13 with loss of function and an acquired von Willebrand syndrome (AVWS).^7,8^ Similar to patients with von Willebrand disease, bleeding complications such as epistaxis and gastrointestinal bleeds are frequent in patients supported with LVAD. This may be due to stimulation of VEGF-dependent vascular proliferation in patients with reduced VWF activity that initiates angiogenesis and encourages the development of arteriovenous malformations.^9^ Additionally, platelets undergo metalloproteolytic shedding of their surface receptors reducing their ability to be activated and to bind to VWF, collagen and fibrinogen.^10^ Thrombocytopenia and hyperfibrinolysis have also been implicated in the bleeding diathesis observed during MCS.^11^

Bleeding is the commonest complication, occurring in up to 60% of LVAD patients and associated with significant morbidity and mortality*.* These patients require frequent blood transfusion leading to sensitisation for alloantibodies and right heart failure, in addition to prolonging hospitalisation which may increase risk of nosocomial infections. Bleeding in the early post-operative period tends to be intervention-related often requiring surgical re-exploration. Gastrointestinal bleeding accounts for the majority of late bleeding events, leading to readmission and its associated complications.^12^

Studies assessing the thrombosis and bleeding complications in patients supported with LVAD have been limited to small, heterogeneous populations and most have excluded patients on prior temporary MCS such as intra-aortic balloon pump (IABP), extracorporeal membrane oxygenation (ECMO) and Impella.^5,10^ These devices are used to bridge the gap to implantation of a durable LVAD or concurrently with LVADs, further aggravating bleeding and thrombosis. Understanding the role of factors associated with adverse events will contribute to the improvement of clinical outcomes through pre-emptive therapy and evidence-based selection of candidates with good prognosis. Therefore, we aimed to assess the incidence of bleeding and thrombosis in patients supported with the HeartWare left ventricular assist device (HVAD), their predictive factors and their impact on 1-year mortality.

## Patients and methods

### Study design and participants

This is a retrospective single-centre observational cohort study in a tertiary cardiothoracic centre in the UK. The study was approved by the Research Ethics Committee, the local Research and Development Office (Reference number: 244283). The need for individual informed consent was waived by the Research Ethics Committee because of the observational nature of the study.

**Figure 1.**
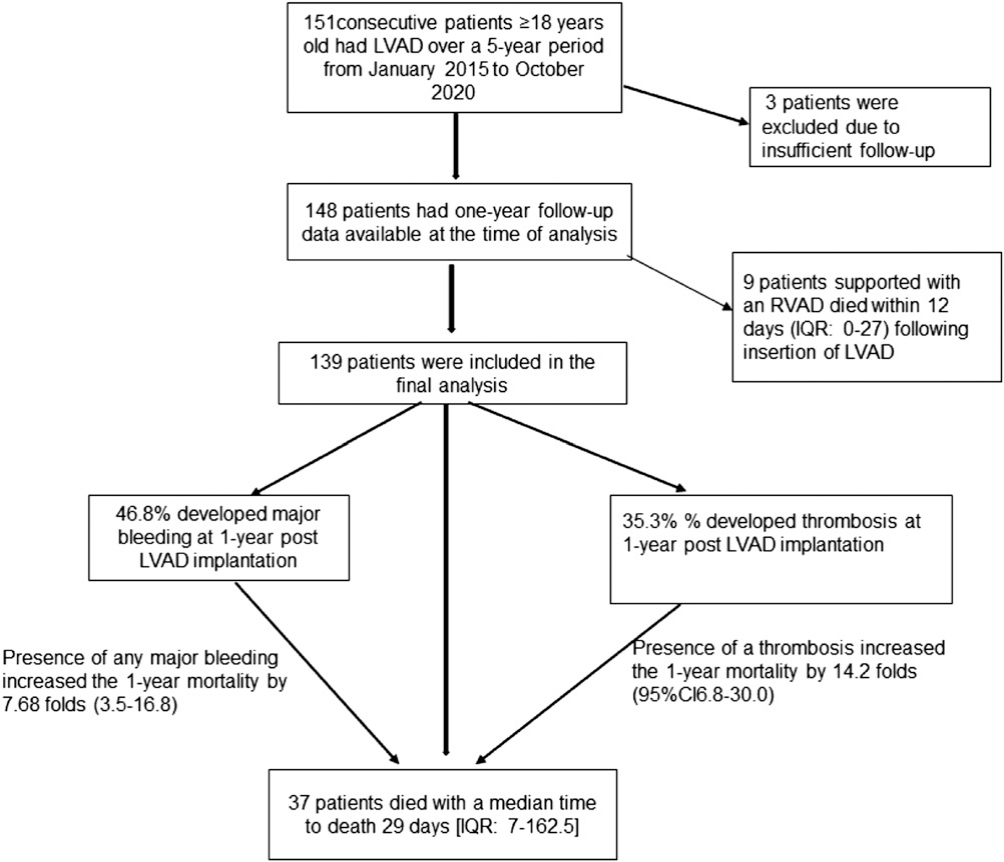
Inclusion and exclusions of patients into the study and overall clinical outcomes within 1-year follow up.

Eligible patients were identified from the Trust LVAD database. We identified 151 consecutive patients ≥18 years old who received a HeartWare LVAD device (HVAD) over a 5-year period from January 2015 until October 2020. Data were collected from the Clinical Data Warehouse, IntelliSpace Critical Care and Anaesthesia (ICCA) and Electronic Patient Records (EPR). Of 151 patients, three patients lacked sufficient follow-up data and therefore were excluded. The remaining 148 patients had one-year follow-up data available at the time of analysis (Figure 1). Patients suitable for LVAD are selected by review at the specialist transplant multidisciplinary meeting at our Trust on the basis that they would otherwise not survive long enough for an organ to be allocated to them on the transplant list. Further details on LVAD insertion are provided in appendix page 1.

### Definitions of outcomes

Primary outcome was survival at one-year post-implantation. Secondary outcomes were bleeding and thrombosis and their impact on survival. Bleeding was defined using the pre-existing Bleeding Academic Research Consortium (BARC)^13^ which standardises definitions of bleeding severity and is summarised in Table S1. BARC grade ≥3 is classified as major bleeding (MB) for this study. Systemic thrombotic events were identified radiologically and classified as either arterial or venous. Venous thrombosis included pulmonary embolism (PE) or deep vein thrombosis (DVT) in the lower limbs or upper limbs or thrombosis in the deep venous system elsewhere in the body. Arterial thrombosis included intracardiac thrombus, ischaemic cerebrovascular incidents, and ischaemic bowel. Device thrombosis was identified clinically according to raised pump pressure, elevated lactate dehydrogenase, elevation of plasma-free haemoglobin >0.3 g/L and raised D-dimer levels in the absence of systemic thrombosis. Infection was defined as positive microorganism culture or clinical suspicion with improvement on antibiotics. Bleeding and infection were classified according to location. Secondary MCS devices were defined as those implanted at the time of LVAD implantation or afterwards. Infections are objectively confirmed using blood cultures with associated raised inflammatory markers such as CRP and PET/CT which has been shown to be very useful in detection of infection long after the device implantation.^14^ We performed PET/CT in LAVD patients: (1) with Bacteraemia; usually during index presentation to differentiate between uncomplicated bacteraemia and device infection or “VAD endocarditis” and after the course of intravenous antibiotics (usually 6 weeks), to decide if they could be discontinued. (2). Complicated driveline infections if there is a suspicion of deeper/central component. (3). Infections from unknown source, especially in the early post-op course, if other imaging tests are conclusive.

### Covariates

Potential predictors of outcomes were collected based on clinical rationale and data availability.

The European System for Cardiac Operative Risk Evaluation II (EuroSCORE II) is a scoring system based on preoperative patient factors, cardiac factors, and operative factors (Table S2). It predicts mortality from cardiac surgery.^15^ Creatinine clearance was calculated from serum creatinine using Cockcroft-Gault Equation. Although optimal transfusion threshold is debatable, haemoglobin ≤70 g/L is the transfusion threshold generally used for our patients. Anaemia was defined as haemoglobin <115 g/L (normal 115–150 g/L) for females and <130 g/L (130–160 g/L) for males based on established laboratory reference ranges. We used the updated EuroSCORE II logistic calculator and in the absence of standardised thresholds, we divided the patients into three categories was based on our data with thresholds: high risk (score ≥ 20%), medium risk (10% < score < 20), and low risk (score < 10%).^15^

### Statistical analysis

Parametric variables are presented as means and standard deviation (SD) whereas non-parametric continuous variables are presented as medians and interquartile ranges (IQR). Probabilities of survival were calculated using the Kaplan-Meir method, and groups compared using the log-rank test. Multivariate survival analysis using a Cox regression model was undertaken to find independently associated prognostic factors. Estimation of bleeding and thrombosis probabilities was undertaken within the competing risks framework, with death the competing risk. Groups were compared using the method of Gray in the univariate setting, and Fine and Gray in multivariate analyses. Significance was set at a *p*-value of 0.05. All analyses were performed using IBM^®^ SPSS^®^ Statistics version 27, or R.

## Results

The baseline characteristics of the 148 patients included in the study are summarised in Table 1. The median age of patients undergoing LVAD implantation was 51.5 years [IRQ 42–61] and 75% were male. Decompensation of heart failure caused by ischaemic cardiomyopathy (31.8%), or non-ischaemic cardiomyopathy (62.8%) were the predominant causes of cardiogenic shock. Other causes included acute myocardial infarction (1.4%), myocarditis (2.7%) and valvular disease (1.4%). Most patients were in advanced heart failure: New York Heart Association (NYHA) III (59%) and IV (13.9%). 63.4% (*n* = 92) of patients had a low risk EuroSCORE II (ESII); 22.8% (*n* = 33) medium risk and 13.8% (*n* = 20) high risk.

**Table 1.** Baseline clinical characteristics and interventions prior to insertion of left ventricular assist devices (LVAD) in 148 patients included in the study.

Age (years) [median (IQR)]	51.5 [42–61]
Gender (%)	
Male	111 (75%)
Female	37 (25%)
BMI (kg/m^2^) [mean (SD)]	27.0 [5.00]
Normal	51 (34.9%)
Overweight	49 (33.6%)
Obese	46 (31.5%)
NYHA	
I	15 (10.4%)
II	24 (16.7%)
III	85 (59%)
IV	20 (13.9%)
Past medical history	
Atrial fibrillation	46 (31.1%)
Previous myocardial infarction	44 (29.7%)
Hypertension	18 (12.2%)
Type II diabetes mellitus	25 (16.9%)
Chronic kidney disease	19 (12.8%)
Dyslipidaemia	15 (10.1%)
Anaemia	12 (8.1%)
Pulmonary hypertension	36 (24.3%)
Thyroid disease	12 (8.1%)
Previous thrombotic event	29 (19.7%)
VTE	12 (8.2%)
STROKE	17 (11.5%)
Coagulation deficiency	2 (1.4%)
FVII	1 (0.7%)
AT	1 (0.7%)
EuroSCORE II (median [IQR])	8.3 [3.6–13]
<100%	92 (63.4%)
10–20%	33 (22.8%)
>20%	20 (13.8%)
Aetiology of cardiogenic shock	
Ischaemic cardiomyopathy	47 (31.8%)
Non-ischaemic cardiomyopathy	93 (62.8%)
Acute myocardial infarction	2 (1.4%)
Valvular heart disease	2 (1.4%)
Myocarditis	4 (2.7%)
Prior MCS device	69 (46.6%)
IABP	20 (13.5%)
VA-ECMO	28 (18.9%)
Impella	12 (8.1%)
RVAD	9 (6.1%)

BMI = Body mass index, NYHA = New York Heart Association; VTE = Venous thromboembolism; FVII = Factor VII, AT = Antithrombin; MCS = Mechanical circulatory support; IABP = intra-aortic balloon pump; VA**-**ECMO = veno-arterial extracorporeal membrane oxygenation, RVAD = right ventricular assist device.

Sixty-nine patients (46.6%) patients required prior temporary MCS. The IABP and VA-ECMO were most commonly (13.5% and 18.9%) used to bridge patients to LVAD implantation.

### Primary outcome: One-year survival

After an initial analysis, prior right ventricular assist device (RVAD) in nine patients was found to be associated with 100% mortality. The median survival for these patients was just 12 days (IQR: 0–27). Eight (88.9%) patients died of multiorgan failure, and one died from infection. These results would heavily influence our analysis of variables associated with adverse events in the greater population of patients who did not receive a prior RVAD. Therefore, subsequent analysis excluded these patients (Figure 1).

For the remaining 139 patients, the probability of survival at 1 year was 73.1%. In total 37 patients died during follow up. The median time to death was 29 days [IQR: 7–162.5] and the leading cause of death was multiorgan failure (*n* = 24). Table 2 summarises univariate analyses of the preoperative variables on one-year survival. Only advancing age and greater ESII score were significantly associated with increased mortality (*p* = 0.007 and *p* < 0.001 respectively), and this remained in the multivariate analysis (Table 2). Patients aged >60 years were twice as likely to die than the younger patients (Hazard ratio (HR) 2.15, 95% Confidence Interval (CI) 1.04–4.47, *p* = 0.038). Also, highest-risk patients with ESII scores >20%, were almost 4-times as likely to die (HR 3.77, 95% CI 1.68–8.48, *p* < 0.0001) than those with scores <10%. The patients with the highest ESII scores were often critical preoperatively, requiring temporary mechanical support or inotropes and had raised pulmonary pressures.

**Table 2.** Univariate and multivariate analyses of preoperative variables for survival at year post LVAD insertion.

	Univariate			Multivariate	
	*N*	Probability of survival at 1 year % (95% CI)	*p*	HR (95% CI)	*p*
Overall	139	76.1			
Gender					
Male	105	72.1	0.72		
Female	34	76.3			
Age (years)					
<60	110	77.9	0.007	1.00	0.038
60+	29	55.2		2.15 (1.04–4.47)	
BMI					
Normal	48	70.6	0.81		
Overweight	47	76.5			
Obese	44	72.4			
Aetiology of cardiogenic shock					
Non-ischaemic cardiomyopathy	45	61.8	0.1		
Ischaemic cardiomyopathy	88	79.4			
Other	6	66.7			
Number of prior devices					
0	101	74	0.2		
1	25	80			
>1	13	51.9			
Prior device: IABP					
No	122	74.4	0.36		
Yes	17	64.2			
Prior device: VAECMO					
No	115	73.6	0.70		
Yes	24	70.8			
Prior device: IMPELLA					
No	129	74.3	0.28		
Yes	10	57.1			
EuroSCORE					
<10%	90	81		1.00	
10–20%	30	69.7	<0.001	1.40 (0.59–3.29)	0.45
>20%	17	41.2		3.77 (1.68–8.48)	<0.0001	

BMI = Body mass index, IABP = intra-aortic balloon pump; VA**-**ECMO = veno-arterial extracorporeal membrane oxygenation, RVAD = right ventricular assist device.

Table 3 summarises the outcomes for patients following LVAD implantation. Nine patients had their LVAD explanted due to successful myocardial recovery (*n* = 1); orthotopic cardiac transplantation (*n* = 5) and exchange due to pump thrombosis (*n* = 3). The median time to explantation was 163 days [IQR: 93–286.5].

**Table 3.** Summary of clinical outcomes in 139 patients had LVAD included in the 1-year follow-up.

Outcomes	*n*	(%)	Median time to event in days [IQR]
All-cause mortality	37	26.6	29 [7–162.5]
Multiorgan failure	25	18.0	26 [7–129]
Bleeding (BARC 5)	5	3.6	88 [17.5–144.5]
Thrombosis	5	3.6	21 [3.5–134]
Unknown	2	1.4	28, 356
Bleeding	65	46.8	14 [2–42]
CNS (ICH)	7	5.0	89 [72–126]
Gastrointestinal	14	10.1	14 [8–108]
Pulmonary	20	14.4	14 [6–32]
Cardiac	12	8.6	1.5 [1–10]
Device site	8	5.7	2 [1–10]
Other^a^	4	2.9	30 [20–149]
Thrombosis	49	35.3	25 [3.5–134]
Venous	12	8.6	29 [2–139]
Arterial	25	18.0	31 [4–175.5]
Device	12	8.6	85 [13–209]
Infection	97	69.8	21 [6–57]
Device related	30	21.6	60 [36–206]
Respiratory	37	26.6	12 [6–41]
Gastrointestinal	2	1.4	5, 324
Genitourinary	5	3.6	25 [14–61]
Other^b^	23	16.5	17 [6–56]
Haemolysis	6	4.3	19 [11–27]
LVAD explant	9	6.4	163 [93–286.5]

CNS= Central nervous system; ICH= Intracranial haemorrhage; LVAD= Left ventricular assist device.

^a^Other bleeding included: epistaxis and haematuria.

^b^Other infection included: infection of unknown origin, infected pressure ulcer.

The incidence of infection (69.8%) surpassed that of any other complication but was not a significant variable for survival. The commonest infections were device-related (driveline) and respiratory with an incidence of 48% and 37% respectively. On average, patients developed an infection 3 weeks post-implantation although device-related infections typically occurred much later at 95 days.

### Incidence of bleeding and thrombosis

Eighty-five patients experienced a bleeding event (61.2%) within 1-year post LVAD implantation. Of these, 20 were BARC grade 1 or 2 and 65 were BARC grade ≥3. Thus, 76.5% of all bleeds were considered major and affected 46.8% of the whole cohort. Early (<3 days) bleeding events were of cardiac and device site origin.

Of the 12 intracranial haemorrhages that occurred, 33.3% (*n* = 4) were fatal and two resulted in profound disability limiting mobility and activities of daily living. Compared to the median time to all-type bleeding, ICH was a late complication typically occurring 3 months post-implantation (median: 89 days, IQR: 72–126). Nineteen patients suffered a gastrointestinal (GI) haemorrhage though only one patient died as a direct result of overwhelming GI bleeding.

Similarly, the incidence of thrombosis was high (49/139, 35.3%). Of these 49 thrombotic events, 12 (24.5%) were venous thrombosis, 25 (51.0%) were arterial thrombosis and 12 (24.5%) device related thrombosis (Table 3). The median time to thrombosis was 25 days (IQR: 3.5–134). All fatal thrombotic events were due to arterial thromboses causing ischaemic stroke (*n* = 4) and one case of ischaemic bowel.

### Impact of major bleeding and thrombosis on one -year mortality

**Figure 2.**
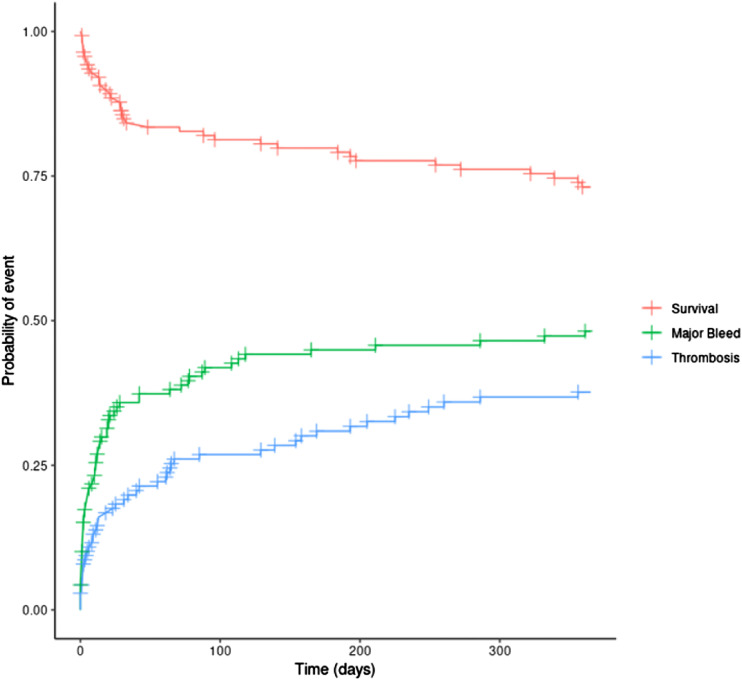
Probabilities of major bleeding, thrombosis, and survival over the 1-year period post LVAD insertion.

The impact of major bleeding and thrombosis on one-year mortality is summarised in Table 4. Overall, bleeding and thrombosis were both significantly associated with mortality after adjusting for patient age and Euroscore in the multivariate analysis. Whilst ‘bleed only’ did not increase the risk of death statistically, patients with any major bleed (with or without a thrombosis) had a 7.68-fold (95% CI 3.5–16.8) increased risk of death compared to those without a MB event. In contrast, patients who had ‘thrombosis only’ had 4.23-fold (95% CI 1.8–10.2) increased risk of death compared those with no thrombosis. The risk of mortality was even higher in patients with any thrombosis (thrombosis alone or in combination of thrombosis and bleeding) whilst the risk of death was highest if the patient had both a major bleed and thrombosis (HR 16.49 [95% 7.7–35.3]). Figure 2 shows the probabilities of MB, thrombosis, and survival over the 1-year period post LVAD insertion.

**Table 4.** Impact of bleeding and thrombosis on 1 year survival following insertion of Left ventricular assist device.

Complication	*N* = (139) (%)	Alive (*n* = 102) (%)	Dead (*n* = 37) (%)	Crude time-adjusted HR(95% CI)	Time-adjusted HR^a^ (95% CI)
No bleed or thrombosis	46 (33.1)	44 (95.7)	2 (4.3)		
Any bleed	65 (46.8)	38 (58.5)	27 (41.5)	7.88 (3.8–16.5)	7.68 (3.5–16.8)
Bleed only	44 (28.1)	32 (72.7)	12 (27.3)	1.90 (0.93–3.9)	1.59 (0.75–3.3)
Any thrombosis	49 (35.3)	26 (53.1)	23 (46.9)	12.76 (6.3–25.8)	14.2 (6.8–30.0)
Thrombosis only	28 (20.1)	20 (71.4)	8 (28.6)	2.82 (1.2–6.4)	4.23 (1.8–10.2)
Bleed and thrombosis	21 (15.1)	6 (28.6)	15 (71.4)	17.84 (8.7–36.4)	16.49 (7.7–35.3)

Major bleeding is defined as >BARC two.

^a^Adjusted for patient age and Euroscore II. All HR’s are shown relative to not having the condition of interest (HR 1.00).

### Factors associated with bleeding and thrombosis

Patients supported by a secondary MCS device were 2.76 times (95% CI: 1.23–6.27, *p* = 0.001) more likely to experience a major bleed. As expected, patients who had major bleeding had 3.46 times (95% CI 1.96–6.10) higher risk of having transfusion of >20 units in the first 7 days of LVAD following LVAD insertion (Table S3).

To assess the impact of early (≤7 days) blood transfusions on bleeding complications beyond the first week post-implantation, we selected only those patients who survived post day 7. When compared to those using fewer transfusion products (<10 units), patients requiring >20 units in the first 7 days were more likely to experience bleeding complications throughout the year (HR 3.46, 95% CI: 1.96–6.10, *p* < 0.0001) (Table S3).

Pre-operative anaemia was significantly associated with an increased risk of thrombosis (HR 3.02, 95% CI: 1.6–5.7, *p* = 0.014) as was use of a secondary MCS device (HR 2.78, 95% CI: 1.2–6.3, *p* = 0.001) (Table S3). Patients receiving aspirin were protected (HR; 0.34 95% CI 0.19–0.58, *p* < 0.001) from all haemostatic complications (bleeding and thrombosis).

## Discussion

In this single centre retrospective study, we investigated the outcomes of 139 patients 1-year post LVAD implantation and identified a potential use of the ESII scoring system in risk stratifying patients preoperatively. Additionally, we observed a significant association between bleeding, thrombosis, and mortality, and identified factors associated with these complications.

In keeping with previous reports, advanced age was strongly associated with mortality.^16^ Even so, the overall mortality has improved from approximately 50%–75% in the era of CF-LVADs despite continued use in those of advanced age.^17^ The association between age and mortality may reflect the multiple comorbidities within this subset of patients. Therefore, identifying the utility of ESII in risk stratifying patients may be important in combining age and gender, comorbidities, pre-operative condition, and operative factors. Menon et al.^18^ compared the use of four scoring systems in predicting outcomes for LVAD patients. In their small cohort of 40, stratifying patients as low risk or high risk according to ESII had a positive predictive value (PPV) for mortality of 0.48 and 0.83 respectively (*p* = 0.004). In comparison, there was no significant difference between the positive predictive value (PPV) of high risk and low risk patients stratified according to the INTERMACS, DTRA, or HMRS at 1 year (*p* = 0.33, *p* = 0.26 and *p* = 0.34).^19^ According to our findings, the ESII scoring system can be used as an independent predictor in assessing the risk of mortality because patients with ESII scores >20% prior to insertion of LVAD had an absolute 3.77-fold risk of 1-year mortality and may have better reproducibility than more subjective scoring systems.

There was a significant association between haemostatic complications and one-year mortality. The incidence of MB was 46.8% (BARC ≥ 3). Cardiac bleeding posed a significant risk of mortality, requiring evacuation which oftentimes would have to be repeated. Balzer et al.^20^ found that incomplete evacuation of cardiac haematomas led to prolonged hospitalisation, and adverse outcomes. Chest bleeding, of cardiac and respiratory tract origin, accounted for the majority of bleeding episodes. Patients with thrombosis, with or without bleeding, and those with combined thrombosis and bleeding complications had the highest risk of 1-year mortality; HR 14.2 (95% CI 6.8–30.0) and 16.49 (95% CI 7.7–35.3) respectively. This demonstrates the difficult balancing act in preventing thrombosis and bleeding in patients supported with LVAD.

Several retrospective studies agree with our finding that high use of early blood products is associated with poor patient outcomes after LVAD implantation (Table 5). Schaffer et al.^21^ identified a significant relationship between intraoperative packed red blood cells (pRBC) and mortality at 1 year with those receiving >5 pRBCs dying at a rate 22% greater than those receiving <5 pRBCs (*p* = 0.05). Over 3 years, Shore et al.^22^ found a 4% increase in mortality for every pRBC unit transfused perioperatively (HR 1.04; 95% CI 1.02–1.07). Contrary to these findings, a small retrospective study by Shrout et al.^23^ observed no association between post-operative transfusion use and one-year outcomes. However, this study of 61 patients used much fewer blood products in the week following implantation. Our findings clarify the relationship between early transfusions and mortality by showing that these patients are at significantly higher risk of continued bleeding.^24^

**Table 5.** Factors associated with bleeding and thrombotic complications in patients with left ventricular assist device Univariate and multivariate analyses of preoperative variables for survival

		N	Probability of outcome (%)	Univariate	Multivariate	
				*P=*	HR (95% CI)	*P=*
Bleeding	Dyslipidaemia No Yes	124 15	34 66.4	**0.027**	0.47 (0.19-1.17)	0.10
	In situ device No Yes	112 27	56.9 88.2	**<0.0001**	2.76 (1.65-4.62)	**<0.0001**
	Week 1 transfusions <10 10-20 20+	57 47 39	44.8 70.1 78.7	**<0.0001**	1.72 (0.99-2.95) 3.46 (1.96-6.10)	0.05 **<0.0001**
Thrombosis	Anaemia No Yes	127 12	35.4 61.9	**0.027**	3.03 (1.61-5.69)	**0.014**
	In situ device No Yes	112 27	32.8 59.2	**0.001**	2.78 (1.23-6.27)	**0.001**

Comorbidities may also compound the risk of thrombosis and anaemia is a well-known risk factor for thrombosis which has previously been associated with adverse outcomes in LVAD cohorts.^25,26^ The prevalence of anaemia amongst patients with advanced heart failure ranges between 30% and 50%,^27^ although LVADs may pose an additional risk. Some post-implantation anaemia may result from erythrocyte damage caused by interactions with artificial surfaces.^28^

Tamrat et al. conducted a retrospective analysis of 205 LVAD patients and of the 34 with post-operative iron deficiency anaemia, the overall risk of ischaemic stroke or device thrombosis was 3.04-times that of non-anaemic patients (95% CI: 1.04–8.8.85, *p* = 0.037).^29^ Population based studies report that anaemia is associated with various thrombotic disorders including myocardial infarction, stroke, and venous thromboembolism (VTE).^30^ Another study on acutely ill hospitalized patients demonstrated that anaemia contributed to a greater risk for symptomatic VTE (2-fold risk of symptomatic DVT or non-fatal PE) through 77 days of follow-up despite the pharmacological thromboprophylaxis.^31^ Chronic inflammation may contribute to anaemia and endothelial dysfunction and blood loss to thrombocytosis, increasing the risk of thrombosis in these patients.^31^ Identifying anaemia before implantation could therefore improve management of these patients.

Finally, employing temporary MCS requires a dynamic approach as patients may be better served by different devices over time. We believe few if any trials have studied specifically the additional risk of haemostatic complications of concurrent use of durable LVADs and other temporary MCS devices. The Impella device is a percutaneous VAD and works similarly to LVADs in facilitating ventricular unloading. Schrage et al.^32^ compared outcomes between patients solely receiving VA-ECMO and those with both an Impella and VA-ECMO (ECMELLA). Indications for ECMELLA were similar to LVAD in our study as it was used to manage patients in cardiogenic shock. ECMELLA was associated with a 21% reduction in 30-days mortality, however this group had a higher incidence of severe bleeding (38.4% vs 17.9%) and ischaemic complications requiring interventions (21.6% vs 12.3%). Despite having been challenged by others,^33,34^ this observation is likely to be true because individual devices are associated with haemostatic complications (Table 1) and concurrent use may exacerbate this. Percutaneous devices also require large vascular access posing an additional bleeding risk.^35,36^ These patients may similarly benefit from increased surveillance, and more specifically a tailored anticoagulation regimen.

Considering these complications, it becomes apparent that clinicians should be vigilant that patients on LVAD support are likely to suffer haemostatic complications. Even in the use of advanced technological devices such as the LVAD, basic clinical examination remains fundamental, particularly auscultation. Muslem et al.,^37^ highlighted its importance in identifying a patient with outflow graft kinking, an LVAD dysfunction that may lead to thrombosis and necessitate replacement.^12^

Furthermore, assessment of haematological parameters is useful for early detection of impending bleeding and thrombotic events. Platelet aggregation tests may help in predicting bleeding associated with platelet dysfunction caused by the devices despite normal platelet count. Additionally, Rahatellah et al. observed that patients with post-implant thrombocytopenia (<150  ×  10^9^/l) were almost five times more likely to bleed.^14^ Similarly, the AVWS results in a loss of large multimers which can be detected using VWF antigen levels (Ag) and ristocetin cofactor activity to calculate the VWF:RCo/VWF:Ag ratio which may be useful in predicting bleeding.^9^ VWF monitoring may also lend itself to the management of LVAD patients receiving blood products. Monitoring of Lactate dehydrogenase (LDH) is useful for early detection of thrombosis or haemolysis. A small study identified a significant increase in LDH in patients experiencing thrombosis (median LDH: 1548 [IQR: 754–2379] vs 363 [IQR: 325–443] U/L, *p*  <  0.0001).^38^ LDH showed high positive predictive value of 88% and a negative predictive value of 97% of identifying haemolysis.^38^

### Limitations

The main limitations of the study are its relatively small sample size and retrospective form. However, all patients were managed using the same institutional anticoagulation and haemostatic management protocol for patients supported with LVAD. We did not include ACT or heparin anti-Xa levels during the LVAD implantation or APTT or heparin level following the surgery in the analysis of bleeding or thrombotic outcomes in this study.

Additionally, HVAD is known to be associated with higher haemostatic complications compared to HeartMate 3™ (HM3) LVAD, a fully magnetically levitated centrifugal-flow pump which was specifically designed to reduce haemostasis-related adverse events including pump thrombosis, strokes, and bleeding.^39^ The pivotal Multicentre Study of MagLev Technology in Patients Undergoing Mechanical Circulatory Support Therapy with HeartMate 3 (MOMENTUM 3) confirmed superiority of the HM3 pump in haemostatic complications, but non-haemostatic adverse events were essentially not different.^40^ Although the usefulness of our study findings may be limited by the withdrawal of HVAD, these data will remain valuable in managing patients with newer LVAD and support devices in general.

## Conclusions

The association with higher bleeding rates emphasizes the importance of perioperative haemostasis in patients supported with LVAD. Bleeding and thrombosis significantly increase the likelihood of mortality and patients with thrombosis with or without bleeding and those with combined thrombosis and bleeding complications had the highest risk of 1-year mortality. This demonstrates the difficult balancing act in preventing thrombosis and bleeding in patients supported with LVAD. Anaemia, multiple in situ devices and high postoperative use of transfusion are also factors which could be modified to mitigate risk. Larger prospective studies are required to validate our findings in newer devices and to risk-stratify patients supported with LVADs.

## Supplemental Material

Supplemental Material - Bleeding and thrombotic complications and their impact on mortality in patients supported with left ventricular assist device for cardiogenic shock Supplemental Material for Bleeding and thrombotic complications and their impact on mortality in patients supported with left ventricular assist device for cardiogenic shock by Ingrid Bekono-Nessah, Alex Rosenburg, Christopher T Bowles, Fernando Riesgo-Gil, Ulrich Stock, Richard R Szydlo, Mike Laffan and Deepa J Arachchillage in Perfusion
