# Supplementary material for: Bleeding and thrombotic complications and their impact on mortality in patients supported with left ventricular assist device for cardiogenic shock
Source: Perfusion. 2022 Sep 23;38(8):1670–81. doi: 10.1177/02676591221127651 (PMC11057213; doi:10.1177/02676591221127651)
Supplement: Supplemental Material - Bleeding and thrombotic complications and their impact on mortality in patients supported with left ventricular assist device for cardiogenic shock [file sj-pdf-1-prf-10.1177_02676591221127651.pdf]

**Supplemenary material: Bleeding and thrombotic complications and their impact on mortality in patients supported with left ventricular assist device for cardiogenic shock**

LVAD implantation

Patients suitable for LVAD are selected by review at the specialist transplant multidisciplinary meeting at our Trust on the basis that they would otherwise not survive long enough for an organ to be allocated to them on the transplant list. All LVADs implanted in the UK are as bridge to transplant (and sometimes bridge to recovery which is demonstrated once implanted) but not as destination therapy which could be the case in other countries

Eligible candidates are booked for LVAD implantation on either an urgent or elective basis. Cardiopulmonary bypass is used to support the native heart throughout the procedure. Afterwards, patients are transferred to the intensive care unit without any anticoagulation. Provided there is no post-operative bleeding, an IV UFH infusion is commenced at 500iu per hour the next morning. The dose is gradually increased to achieve a heparin anti-Xa level of 0.2-0.4 and eventually converted to warfarin with a target international normalized ratio (INR) of 2.5 (2.0-3.0) before discharge[11]. After discharge, patients are followed up by the VAD team and are monitored at outpatient clinics. As per our protocol, it is the standard practice that all patients with LVAD are managed with dual therapy: warfarin and aspirin, unless contraindications (mainly history of life-threatening or refractory bleeding).

If a patient develops major bleeding, anticoagulation is withheld for a period depending on the site and the severity of the bleeding. In patients with intracerebral bleeding anticoagulation is withheld for 7-10 days and reimaging is performed to assess the progression of the bleeding prior to re-starting the anticoagulation. Major bleeding at other sites is managed by either withholding anticoagulation for 24-48hrs or reducing the intensity of anticoagulation on individual patient basis. Patients with clinically significant non-major bleeding are generally managed by reducing the intensity of anticoagulation until the bleeding has settled with or without 24-48hrs of tranexamic acid. Patients who develop thrombosis whilst on standard anticoagulation

Formatted: Font: (Default) Arial, No[illegible] Italic

intensity are managed by increasing the intensity of anticoagulation i.e increasing the INR target from 2.5 to 3.0 with or without adding second antiplatelet treatment depending on the individual patient's risk of thrombosis vs bleeding. All patients who develop major bleeding or thrombosis are discussed with consultant haematologists and management plan is agreed on a case-by-case basis.

**Table S1: Bleeding and academic consortium (BARC) classification used to classify clinical severity of bleeding episodes. This table has been taken from the BARC guidelines.**

| | |
|---|---|
| **Grade 0** | No bleeding |
| **Grade 1** | Bleeding that is not actionable and does not cause the patient to seek treatment |
| **Grade 2** | Any clinically overt sign of haemorrhage that "is actionable" and requires diagnostic studies, hospitalization, or treatment by a health care professional but does not meet criteria for grade 3,4 or 5 |
| **Grade 3** | a. Overt bleeding plus haemoglobin drop of 3 to < 5 g/dL (provided haemoglobin drop is related to bleed); transfusion with overt bleeding<br><br>b. Overt bleeding plus haemoglobin drop > 5 g/dL (provided haemoglobin drop is related to bleed); cardiac tamponade; bleeding requiring surgical intervention for control (excluding dental/nasal/skin/haemorrhoid); bleeding requiring IV vasoactive agents.<br><br>c. Intracranial haemorrhage confirmed by autopsy, imaging, or lumbar puncture; intraocular bleed compromising vision |
| **Grade 4** | CABG-related bleeding within 48 hours<br><br>-Perioperative intracranial bleeding within 48 hours<br><br>-Reoperation after closure of sternotomy for the purpose of controlling bleeding<br><br>-Transfusion of ≥5 U whole blood or packed red blood cells within a 48-hour period<br><br>-Chest tube output 2 L within a 24-hour period<br><br>Notes: If a CABG-related bleed is not adjudicated as at least a type 3 severity event, it will be classified as not a bleeding event. If a bleeding event occurs with a clear temporal relationship to CABG (ie, within a 48-hour time frame) but does not meet type 4 severity criteria, it will be classified as not a bleeding event. |
| **Grade 5** | a. Probable fatal bleeding<br><br>b. Definite fatal bleeding (overt or autopsy or imaging confirmation) |

**Table S2: Factors included in the EuroSCORE II logistic calculation to predict operative mortality after cardiac surgery.**

| **Patient factors** | **Cardiac factors** | **Operative factors** |
|---|---|---|
| Age (years) | New York Heart Association (NYHA) | Urgency |
| Gender | Unstable angina | Weight of the intervention |
| Renal impairment (creatinine clearance) | Left ventricle ejection fraction (%) | Surgery on thoracic aorta |
| Extracardiac arteriopathy | Recent MI (<90 days) | |
| Poor mobility | Pulmonary hypertension<br>[PASP (>31mmHg)] | |
| Previous cardiac surgery | | |
| Chronic lung disease | | |
| Active endocarditis | | |
| Critical preoperative state | | |
| Diabetes | | |

**Table S3**: Factors associated with bleeding and thrombotic complications in patients with left ventricular assist device Univariate and multivariate analyses of preoperative variables for survival

| | | N | Probability of outcome (%) | Univariate | Multivariate | |
|---|---|---|---|---|---|---|
| | | | | *P*= | HR (95% CI) | *P*= |
| **Bleeding** | Dyslipidaemia | | | | | |
| | No | 124 | 34 | **0.027** | | |
| | Yes | 15 | 66.4 | | 0.47 (0.19-1.17) | 0.10 |
| | In situ device | | | | | |
| | No | 112 | 56.9 | **<0.0001** | | |
| | Yes | 27 | 88.2 | | 2.76 (1.65-4.62) | **<0.0001** |
| | Week 1 transfusions | | | | | |
| | <10 | 57 | 44.8 | **<0.0001** | | |
| | 10-20 | 47 | 70.1 | | 1.72 (0.99-2.95) | 0.05 |
| | 20+ | 39 | 78.7 | | 3.46 (1.96-6.10) | **<0.0001** |
| **Thrombosis** | Anaemia | | | | | |
| | No | 127 | 35.4 | | | |
| | Yes | 12 | 61.9 | **0.027** | 3.02 (1.61-5.69) | **0.014** |
| | In situ device | | | | | |
| | No | 112 | 32.8 | | | |
| | Yes | 27 | 59.2 | **0.001** | 2.78 (1.23-6.27) | **0.001** |
